# A randomized pilot trial of growth hormone with anastrozole versus growth hormone alone, starting at the very end of puberty in adolescents with idiopathic short stature

**DOI:** 10.1186/1687-9856-2015-4

**Published:** 2015-02-16

**Authors:** Anya Rothenbuhler, Agnès Linglart, Pierre Bougnères

**Affiliations:** Department of Pediatric Endocrinology, Bicêtre Hospital, Pôle I3E, AP-HP, Paris Sud University, 94275 Le Kremlin Bicêtre, France

**Keywords:** Idiopathic short stature, Anastrozole, Growth hormone, Short children, End of puberty

## Abstract

**Background:**

When given during the course of puberty, anastrozole (A), an aromatase inhibitor, has been shown to increase the predicted adult height (PAH) of GH-deficient (GHD) boys treated with recombinant human growth hormone (rhGH). Our study questioned whether this treatment could retain some of its effects in non-GHD adolescent boys if started only at the very end of puberty, a time when rhGH treatment is denied to short adolescents who have almost reached their final height.

**Objective:**

To explore the effect on adult height of a combination of rhGH and A, compared with rhGH alone, at the end of puberty in boys with idiopatic short stature (ISS).

**Methods:**

A prospective randomized study comparing rhGH + A and rhGH was conducted in 24 healthy adolescent boys aged 15.2 ± 1.2 yrs with serum testosterone at adult levels and a faltering growth velocity <3.5 cm/yr leading to a predicted adult height (PAH) <2.5 SDS. Treatments were stopped when growth velocity became <10 mm in 6 months or when height was close to 170 cm. A historical group of ISS adolescents (N = 17) matched for puberty and growth was used for comparison.

**Results:**

IGF1 levels remained within normal limits in all treated patients. Mean treatment duration was 19 months in the rhGH + A group and 11.5 months in the rhGH group (P = 6.10^−4^). Adult height reached 168.4 ± 2.6 cm in the rhGH + A group and 164.2 ± 5.6 cm in the rhGH group (P < 0.02). Adult height was 160.1 ± 2.8 cm in the historical controls.

**Conclusion:**

A combination of rhGH and A, started at the very end of puberty, seems to allow boys with ISS to reach a greater adult height than rhGH alone. Larger trials are needed to confirm this preliminary observation.

**Electronic supplementary material:**

The online version of this article (doi:10.1186/1687-9856-2015-4) contains supplementary material, which is available to authorized users.

## Introduction

Idiopathic short stature (ISS) describes a heterogeneous group of children of unknown etiology [1–4] who become adults of short stature [5–18]. Based on general considerations on the tolerability of short stature by adults [19–30], and on the limited height benefit that is considered to result from years of a costly treatment whose long term safety has been questioned (see Discussion) [31–37], the use of recombinant human growth hormone (rhGH) to increase the height of healthy children with ISS remains debated. The prerequisites for the use of rhGH in ISS set by the FDA are that other diagnoses are excluded, that the presenting height is < −2.25 SDS for age and sex, and that adult stature is expected to be < −2.0 SDS [2]. Several reviews of studies on treatment with rhGH in ISS [1–3, 38–40] concluded that a mean gain in predicted adult height (PAH) of ~5-7 cm can be expected following an average of 5.4 years of treatment. More meaningful information comes from studies that have provided adult height values [12–18, 41–44]. In fact, the different studies showed different rhGH-induced height gains [5, 12–18, 41–52], for reasons that are most clearly discussed in the study by Sotos and al [41]. The potential growth promoting effect of starting rhGH administration at the very end of puberty, months to years after height peak velocity is passed, has not been explored yet. At his particular moment, the fusion of epiphyseal plates of the long bones governs the tempo of growth deceleration; when growth velocity falls under 15 mm per 6 months, cessation of growth is expected to occur within the next two years [53–56].

The use of aromatase inhibitors for promoting growth has been recently reviewed [57–63] and a debate has started in the pediatric endocrinology community regarding the benefit/risk ratio of these drugs [64, 65]. In non- growth hormone deficient (GHD) boys with ISS and/or delayed puberty, aromatase inhibitors effectively delay bone maturation and thereby increase PAH [66–69]. In a Finnish study, 23 boys aged 15.1 years with delayed puberty were randomly allocated to 1 year of letrozole or placebo. Both groups also received testosterone injections for 6 months and were evaluated 18 months after initiation of therapy [66]. A third, nonrandomized group received no treatment. PAH increased by 5.1 cm with letrozole vs 0.3 cm with placebo. The nonrandomized, untreated controls gained 2 cm. In a follow-up study [68], the near-adult height of the letrozole-treated group was 6.9 cm more than the placebo group, positive results that might have been affected by a selection bias at start of treatment [57]. In another 2-year randomized study of 91 Iranian boys with a constitutional delay of growth and puberty, letrozole increased PAH more than placebo [69]. In a Finnish study, 30 boys with ISS aged 9.0–14.5 years were randomly allocated to receive either letrozole or placebo for 2 years [67]. Most participants (81% and 93%, respectively) had not entered puberty at the start of the study, and 44% after 2 years. Height at start was < −2 SDS and mean bone age < 14 years. Letrozole-treated boys showed growth velocities similar to those receiving placebo, and again bone age advanced less with letrozole therapy, thus the PAH increased by 5.9 cm However, when reevaluating the results in 23/30 six years after starting the study, the difference in PAH was no longer statistically significant (166.5 cm in letrozole-treated versus 162.4 cm in placebo-treated) [70]. Adult height data are not available for these trials [57].

The efficacy of anastrozole co-treatment with rhGH has been investigated in GHD boys in two studies from the same US center [71, 72]. An open-label pilot study on 20 patients treated for 1 year did not show an effect on PAH [71]. In a later study, 52 male adolescents treated with rhGH for GH deficiency were randomly allocated to co-treatment with A or placebo for 1–3 years [72]. At entry, serum testosterone was in a 1–3 ng/ml range. PAH increased in the A-treated group by 6.7 cm after 3 years, whereas only 1 cm of PAH gain was observed in the placebo group [72]. The decrease in growth velocity during the course of the study was greater in the placebo group than in the A group at 36 months [61, 72] No adult height data are available to date.

**Figure 1:**
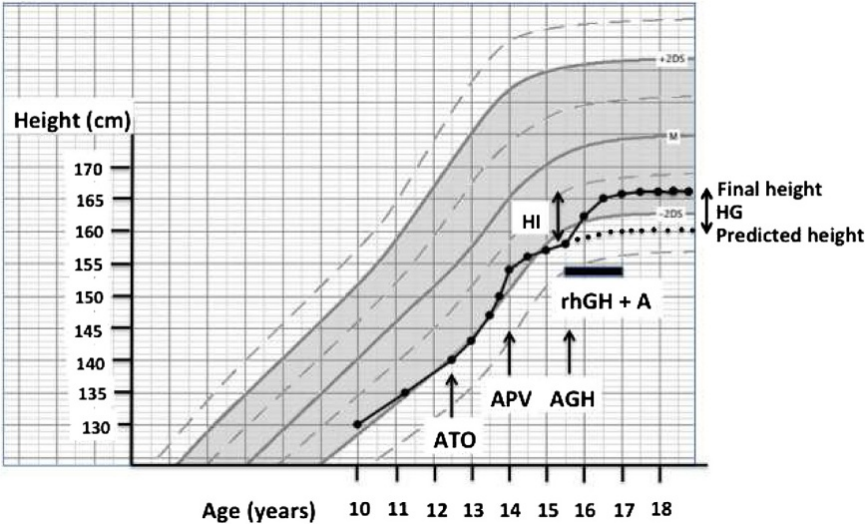
**Pubertal growth and response to rhGH + A treatment in a representative adolescent.** Height of a patient showing actual growth (continuous dotted line), age at take off (ATO), age at peak growth velocity (APV), age at rhGH onset, predicted growth trajectory, response to GH administration and final height. HG = height gain. HI = height increase.

These results paved the way for testing the combination of rhGH + A in adolescents with ISS who are finishing their growth. Indeed, we were not aware of any trial having tested the combination of rhGH and A in ISS during the late stage of near-ending growth, likely because adolescents, families, and most pediatric endocrinologists believe it would be too late for rhGH to allow a significant gain in stature. The current pilot trial questioned this belief in a sample of adolescents with predicted adult short stature. When their growth velocity drops, boys of short stature realize that they will not be able to reach an adult height acceptable for them (see an example in Figure 1).

## Methods

### Patients

Based on previous results with aromatase inhibitors, a minimal sample size was calculated to allow detecting a 6 cm increase in final height by adding A to rhGH with an alpha of 0.05 and a power of 0.80. We included 24 adolescents who consulted us between March 2004 and September 2009. They were selected on the following inclusion criteria: a complete or near complete sexual maturation (testes volume >12 ml and adult testosterone levels), a reliable series of height measurements over puberty showing a decelerating growth velocity equal or less than 3.5 cm/yr during the preceding 6 months, leading to a PAH < −2.5 SDS (see our model for calculation in lower section). We chose this height because a well-conducted quality-of-life (QOL) study set 160 cm as the threshold for observing a negative impact of short stature on male adults [19]. Although only a fraction of these adolescents fulfilled the strict definition of ISS at time of entry (because their height was not < −2SDS), they would have fulfilled the definition at adult ages, given the PAH. Although a mix of short stature and mildly advanced puberty would seem a more accurate definition for approximately half of the adolescent boys, we used the generic term “ISS” to define their category for simplification. GH deficiency was excluded by a stimulation test with a GH peak value superior to 15 ng/ml. This cut-off threshold was chosen instead of the usual 5–10 ng/ml value to adjust to the age of the participants and ensure that no studied adolescent had any degree of GH deficiency. Subtle forms of dyschondrosteosis or other chondrodysplasia were excluded by radiographs of the forearm, spine, hand, pelvis and leg. TSH levels were normal. All adolescents were healthy.

The growth trajectory could be modeled in the adolescents from their “Carnet de Santé”, a national pediatric health booklet where height and weight are reported by pediatricians or general practitioners during the period of growth [73]. The pubertal growth spurt trajectory was approximated using the height and age at the two inflection points that mark the onset of acceleration (take-off) and starting deceleration (peak growth velocity), respectively (Figure 1 and Tables 1 and 2) [53, 54]. Height measurements were performed independently at onset and end of visit, using a stadiometer with 0.11% precision (SD/mean). Testes volume was evaluated with a direct measurement of their major and minor axes and calculation of the corresponding ellipsoid.

**Table 1:** **Characteristics of the 24 adolescent males at onset of rhGH or rhGH + Anastrazole administration and historical controls**

	GH alone	GH + Anastrazole	Historicals controls
N	*12*	*12*	*17*
Father’s height			
cm	168.5 ± 4.9	168.9 ± 3.6	169.2 ± 4.1
SDS	- 1.2 ± 0.8	- 1.1 ± 0.6	−1.1 ± 0.8
Mother’s height			
cm	157.8 ± 5.8	156.4 ± 6	157.9 ± 5
SDS	- 1.2 ± 1	- 1.2 ± 1.1	−1.2 ± 1
Birth length (cm)	48.3 ± 1	48.2 ± 1.2	48.4 ± 1.2
Age (yrs)	15.2 ± 1.1	15.2 ± 0.8	15.1 ± 0.8
Height			
cm	156.3 ± 2.9	155.9 ± 4	156.1 ± 3.5
SDS	- 1.70 ± 1	- 1.70 ± 0.7	−1.67 ± 0.8
Weight (kg)	49.8 ± 4	48 ± 6	49.9 ± 4
Testes volume (ml)	22.4 ± 8	22.2 ± 5	22 ± 5
Serum testosterone (ng/ml)	5.5 ± 0.9	5.6 ± 0.9	5.5 ± 0.8
Bone age at hand (yrs)	14.6 ± 0.6	14.5 ± 0.8	14.6 ± 0.7
Knee score*	2.8 ± 0.7	2.8 ± 0.4	2.7 ± 0.4 (N = 10)
IGF1 (SDS)	- 0.5 ± 0.4	- 0.3 ± 0.6	−0.4 ± 0.6 (N = 10)

We observed no significant differences between the groups. Values are expressed as mean ± SD.

*see Methods.

**Table 2:** **Characteristics of the growth curve of the 24 adolescent males at onset of rhGH or rhGH + Anastrazole administration**

	GH alone	GH + Anastrazole	Historical controls
N	*12*	*12*	*17*
Age at take-off of growth (yrs)	12.3 ± 1.2	12.3 ± 0.9	12.2 ± 1.5
Height at take-off of growth			
cm	139.7 ± 4	139.7 ± 3	140 ± 5
SDS	- 1.2 ± 0.7	- 1.5 ± 0.9	−1.2 ± 0.9
Age at peak velocity (yrs)	14.3 ± 1	14.2 ± 0.7	14.3 ± 1.2
Height at peak velocity (HPV)			
cm	154.1 ± 4	153.6 ± 5	154 ± 6
SDS	- 1.3 ± 1	- 1.1 ± 0.8	−1.3 ± 1.1
Time interval between PGV and GH onset (mo)*^a^	0.9 ± 0.6	1 ± 0.5	1 ± 0.7**
Growth velocity between PGV and GH onset			
cm/yr	2 ± 1	2.3 ± 0.7	2.2 ± 0.8**
SDS	- 2.6 ± 1.4	- 3 ± 1.7	−2.8 ± 1.9**

We observed no significant differences between the groups. Values are expressed as mean ± SD.

^a^*see Methods.

**mean age at GH onset in the GH treated adolescents was used to calculate corresponding values for time interval and growth velocity in the historical controls.

The same investigators (GK, AR, PB) evaluated bone age at the hand independently according to Greulich and Pyle [74] and the degree of closing of the femoral inferior and tibial superior growth plates according to O'Connor and Roche [75, 76] then averaged their estimations. These evaluations were blind to treatment allocation.

The parents were informed by written material about the uncertainties of rhGH or A benefits at this age, about the results of the rhGH and A safety studies, and knew the position of the national agencies before giving their written informed consent to the trial according to the French rules of bioethics.

A group of historical controls was formed with 17 untreated adolescent males with ISS matched for testosterone, bone age and growth velocity (3 were older brothers of adolescents from the rhGH group, 3 from the rhGH + A group). These subjects have consulted our center for ISS without a strong enough motivation for considering GH treatment. We used the data collected from their Carnet de santé and final height to model their growth trajectory. Knee score was available in only 9 of them. Other missing data include a precise following of growth trajectory at the end of adolescence and serial IGF1 measurements

### Randomization and treatment protocol

The randomization procedure was chosen to allow the parallel treatment of participating children under each regimen. Initially, our three-arm protocol randomly allocated 1 subject per group of 3 adolescents (allocation was drawn at pre-inclusion in the trial) to rhGH, rhGH + A, or no treatment. After three years and inclusion of six groups (N = 18), a primary refusal had occurred in 4/6 subjects in the untreated group, who sought rhGH treatment in other centers. Therefore we could not maintain a reasonable rate of accrual for the recruitment of the untreated arm, thus we switched our randomization process to allocation of 2 subjects within groups of 4 to rhGH or rhGH + A treatment. We stopped the inclusions after a total of 12 subjects had been included in each treatment arm.

rhGH was given at an initial dose of approximately 0.07 mg/kg.d then adjusted to growth velocity while maintaining serum IGF1 level close to +1SDS. Anastrozole (A) was given at a daily dose of 2 mg. The doses of both rhGH and A were deliberately chosen to be high, based on the short expected duration of trial, in order to maximize the therapeutic effects.

Patients were seen every 3 months until near-end of growth. Treatment was stopped when growth velocity under rhGH treatment was < 10 mm over 6 months (N = 18) or when the adolescent has reached a height near 170 cm (N = 6).

At each consultation, parents and adolescents were asked to fill out a questionnaire that listed known secondary effects of A, including mood changes and neuro-psychic symptoms (depression, nervousness, dizziness, insomnia, weakness), hot flashes, digestive symptoms (stomach pain, nausea, loss of appetite, constipation, diarrhea, vomiting), skin (rash, acne), joint symptoms (arthralgia, arthritis), back pain, muscle pain, headaches.

### Biological parameters

Serum IGF1 levels were measured between 7 and 11 am, 12–16 hours after the previous evening rhGH injection. Values at 6, 9, 12, 15, 18 and 24 months were used for monitoring rhGH treatment and were averaged to calculate individual IGF1 means during rhGH administration Serum IGF1 was measured by immunoradiometric assay after ethanol-acid extraction using DSL-5600 Active reagents (Diagnostic Systems Laboratories, Webster, TX). IGF1 SDS calculations were provided by DSL as reported [77]. Intra- and interseries coefficients of variation were 1.5 and 3.7% at 260 ng/ml, and 2.5 and 3.9% at 760 ng/ml. The sensitivity was 4 ng/ml. FSH and LH were measured as reported by a time-resolved fluorometric assay using Delfia reagents (Perkin Elmer Life Sciences, Courtaboeuf, France). Sensitivity for both assays was 0.01 IU/liter. Serum testosterone was measured every six months with a direct RIA (CisBio International, Gif sur Yvette, France). Sensitivity was 0.01 ng/ml (0.05 nmol/liter).

### Growth model, calculation and statistics

As pointed by Garn et al. [78] the bone age evaluated at the hand (wrist and phalanges) at the end of puberty does not predict the complete epiphyseal union of long bones of the leg, thus we did not use the Bayley Pinneau method based on the hand. Instead, we modeled the deceleration of growth velocity in each adolescent to be able to extrapolate adult height, using the age (t) and height (h) of each subject accurately measured during the deceleration period, we modeled. We considered that growth velocity thereafter slows uniformly and is terminated within 2 years from the peak growth velocity. The slope p of the curve is approximated from the recorded values at peak growth velocity (t1,h1) and at rhGH onset(t2,h2) as p = (h2-h1)/(t2-t1)). The derivative between rhGH onset and final h is f'(t) = p - p/(t_fin_-t2) * (t-t2). Integral is f(t) = p*t - p/(t_fin_-t2)* (1/2*t^2^ - t2*t) + K. When t = t2, f(t2) = h2, then k = h2 - {p*t2 + p/2(t_fin_-t2)*t2^2^}. The growth curve is thus made of real values between t1 and t2, then between t2 and t_fin_ of the function that we determined, then becomes null 2 years after peak growth velocity time. We tested the potential benefit of rhGH + A treatment vs rhGH treatment with the Student’s t test and the chi-square test.

## Results

The two randomized groups of adolescent boys had comparable characteristics as shown in Table 1 including the pubertal growth spurt trajectory shown in Table 2. None of the studied adolescents had a constitutional delay of puberty, 18/22 had followed a normal maturation pattern. At inclusion, four could be considered early maturing boys according to Sandberg and al [23], with ages at take-off growth 10–12 yrs, age at peak growth velocity 12.5-13 yrs and full sexual development achieved at age 13–13.5 yrs. rhGH treatment was initiated at 15.2 ± 1.2 yrs in the rhGH group and 15.2 ± 0.8 yrs in the rhGH + A group.

The main parameters of the treatment and height evolution are shown in Table 3. A representative growth chart (Figure 1) illustrates what we observed in many children. The 12 adolescents of the rhGH group stopped growing after 11.5 ± 5 months, while those in the rhGH + A group kept growing for 19 ± 6 months (P = 6.10^−4^), leading to termination of treatment at 16.2 ± 1.1 yrs in the rhGH group and 16.8 ± 0.6 yrs in the rhGH + A group. During the treatment period, all patients maintained their IGF1 level within 0–1.5 SDS (average 0.60 ± 0.45 SDS). Only 5 of the 66 total IGF1 measures exceeded +2SD on one occasion and not one IGF1 value ever exceeded +2.5 SDS.

**Table 3:** **Main parameters and effects of of rhGH or rhGH + Anastrazole administration in the 24 adolescent males**

	GH alone	GH + Anastrazole	P
N	*12*	*12*	
GH dosage (mg/k.d)	0.073 ± 0.09	0.076 ± 0.01	*NS*
Duration of GH administration (mo)	11.5 ± 5	19 ± 5.9	0.02
Age at end of GH (yrs)	16.2 ± 1.1	16.8 ± 0.7	0.08
Mean IGF1 (SDS)	0.55 ± 0.3	0.65 ± 0.6	*NS*
Delta IGF1 (SDS)*	1.02 ± 0.3	0.97 ± 0.5	*NS*
Predicted height**			
cm	158.2 ± 2.9	157.9 ± 3.8	*NS*
SDS	- 2.84 ± 0.5	- 2.9 ± 0.6	*NS*
Final height			
cm	164.2 ± 5.6	168.4 ± 2.6	0.02
SDS	- 1.8 ± 0.9	- 1.1 ± 0.4	0.02
Growth velocity Year 1 (cm/yr)	4.9 ± 4	6.7 ± 2.8	0.18
Growth velocity Year 2 (cm/yr)	1.2 ± 0.5	3.6 ± 2.7	0.008
Height increase from GH onset (HtG_1_) (cm)	7.8 ± 5	12.7 ± 5.6	0.02
Height gain vs PAH (HtG_2_) (cm)	5.9 ± 4.5	10.5 ± 5.2	0.02

Values are expressed as mean ± SD.

*Mean IGF1 during GH-IGF1 at onset of GH.

**see Methods/Calculation.

Adult height was 164.2 ± 5.6 cm in the rhGH alone group and 168.4 ± 2.6 cm in the rhGH + A group (P = 0.019). The difference between adult height and height at rhGH onset was 7.8 ± 5 cm in the rhGH group, and 12.7 ± 5.6 cm in the rhGH + A group (P = 0.023). Individual height increase was variable. For example, 4/12 (33%) adolescents in the rhGH group showed an increase of height <3 cm (mean 1.85 cm, range 0.6 to 2.6 cm) vs 0% in the rhGH + A group, despite comparable knee score and growth velocity at onset of treatment (P = 0.046).

**Figure 2:**
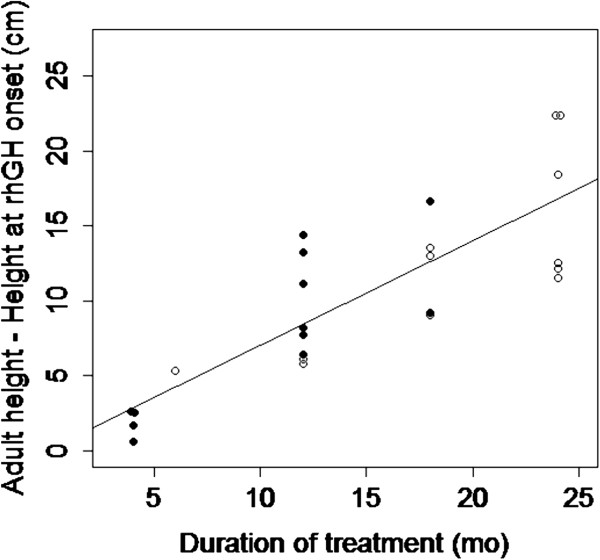
**Correlation between the duration of rhGH administration and height increase.** Black dots figure the children treated with rhGH alone, empty circles those treated with rhGH and anastrozole. The height increase up to final height is closely correlated with the duration of rhGH administration according to the equation Y = 70X + 0.025 (R = 0.82, P = 2×10^−7^).

We found that the increase in height correlated closely with the duration of rhGH treatment (R = −0.82, P = 0.01) (Figure 2). The magnitude of the height gain was also negatively correlated with the knee score when both groups were merged (R = −0.59, P = 0.002), not with bone age at the hand. In fact, the correlation of height increase with knee score was strong in the adolescents treated with rhGH alone (R = −0.86, P = 0.01). We also found that the persisting growth velocity at the time of rhGH onset was another predictor of height increase (R = 0.70, P = 0.0015). The failure to respond to rhGH was associated with a higher knee score and a minimal growth velocity at time of rhGH onset.

We found no significant correlation of height increase with baseline IGF1 or with the increase in IGF1 in response to rhGH, nor with other studied parameters in either group or the two merged groups listed in Additional file 1 and Additional file 2.

At entry in the trial, PAH calculated with our equation is 158.2 ± 2.9 cm in the rhGH group and 157.9 ± 3.7 cm in the rhGH + A group. Therefore the difference between reached adult height and PAH before treatment was 5.9 ± 4.5 cm in the rhGH group and 10.5 ± 5.1 cm in the rhGH + A group (P = 0.019). This difference had the expected high degree of correlation with the increase in height from the onset of rhGH administration (Y = 1.0X + 1.8; R = 0.97, P = 5.10^−9^), and the same predictors (Additional file 1).

The historical controls were comparable to the participants for age, bone age at the hand and epiphyseal closure at knee, testes volume and testosterone levels at entry into the trial (Tables 1 and 2). They reached a mean final adult height of 160.1 ± 2.8 cm (−2.5 SDS), lower than that of adolescents treated with rhGH (P < 0.05) or with rhGH + A (P < 0.0001) (Table 3). This statistical difference should however be considered questionable given the non-randomized nature of the untreated group. True adult height was close to PAH (calculated to be 160.3 ± 5 cm), which validates our predictive equation.

We detected no significant secondary effects of rhGH or A during the short observation period (Additional file 2). Lipid values, testosterone and gonadotropin values during the rhGH + A treatment are presented at Additional file 3.

## Discussion

rhGH studies on ISS have focused on childhood or on the beginning of puberty [2, 7, 12–18, 20, 33, 37–52], with the belief that a younger age favors rhGH effects [37]. Growth-promoting effects of aromatase inhibitors, employed alone [66–70, 79, 80] or in conjunction with rhGH [71, 72], have only been explored in pre-pubertal adolescents with ISS [67, 70, 79] or in adolescents with constitutional delay of puberty [66, 68–70] or GH deficiency [71, 72]. Despite an extensive Pubmed search, we were not able to find a single study that tested the effect of rhGH and/or aromatase inhibitors starting during the latest stage of puberty in adolescents with ISS.

### Assessment of Benefit and Cost

Using adult height as a gold-standard outcome in growth studies, the current observation suggests that combination of rhGH and A allows for an additional mean height gain of 4.9 cm versus rhGH alone, with a large variability of individual responses. The main predictor of height gain was the duration of treatment, which was longer by 7.5 months in the rhGH + A group. The other predictors of height gain were the knee score, not the largely used hand bone age, and the remaining growth velocity at rhGH onset.

A weakness of our trial is the lack of a true control group. We could only compare the adult heights of our patients with PAH calculated by an equation especially designed for near-ending and decelerating growth. Since this equation proved capable of predicting adult height accurately in historical controls, we felt comfortable to use it for the treated patients. We found that rhGH allowed a height gain close to 6 cm versus PAH, and that addition of A to rhGH may have increased this gain up to 10.5 cm in average. Comparison with non-randomized controls suggests a gain in final adult height of 4 cm with rhGH alone and 8.3 cm with rhGH + A. In summary, the rhGH + A treatment seems capable of increasing the adult height of adolescent boys with ISS by 8–10 cm. We stress however that the latter values should only be considered indicative until randomized controlled trials in ISS adolescents allow a more reliable estimation of the height gain improvement.

The optimal dose and duration of rhGH treatment are debated in children with ISS. The impression is that despite the significant gain in height, many rhGH-treated children remain short as adults, in the lower level of the normal range. This may simply be that most studies have used rhGH doses of 0.16 to 0.26 mg/kg/week, which may not have been adequate [41]. Several dose–response studies in prepubertal children with ISS have explored a wide range of rhGH doses from 0.20-1.75 mg/kg/week [2, 12, 17, 38–40, 43, 50–52, 77]. The benefit obtained seems dose dependent and mean benefits of 7–8 cm for adult height have been reported with doses of 0.32 to 0.4 mg/kg/week [11, 12, 17] consistently with a recent report from the US [41]. Given that the treatment duration was expected to be short in our trial, we selected a rhGH dose around 0.5 mg/kg/week then guided therapy using growth velocity and IGF1 levels. The total rhGH dose delivered to the adolescents with ISS was 21 mg/kg in the rhGH group and 35 mg/kg in the rhGH + A group, respectively. Although given at a higher weekly dosage, these cumulative doses represent only 23% and 38% of the 91 mg/kg totaled by the majority of children with ISS who are treated with a traditionally recommended dose of 0.05 mg/kg.d for an average of 5.4 yrs [2]. Although this is yet speculative, it is thus possible that our regimen of IGF1-based rhGH dosing may offer a dose-sparing and safer mode of therapy, as discussed by Chen and al [51]. Clearly however, it would be premature to draw conclusions from such a small number of children on trial, who are being presented here to stimulate the study of new rhGH regimens for short stature. The intention of our trial was initially to find a mean to rescue short stature at a time of near finished growth. Notably, our approach of rhGH + A administration at the end of puberty cannot be extrapolated to a large proportion of children with ISS before comparison is performed with the traditional prolonged rhGH treatment at lower classical doses starting at younger ages through larger trials.

The cost to the patients (or insurances), for the purchase of rhGH from distributing pharmacies, may be as much as $88,000 to $100,000 per gram in the USA [41]. If our observations are confirmed in larger series of adolescents with ISS, they might contribute to design a cheaper treatment of ISS without compromising height gain. The estimated cost of rhGH therapy compared with no therapy, in 2011, was $47,000 or more per cm [72], depending on unit cost and height gain. If our results were confirmed, the 8–10 cm gain would translate into a cost of $18,000 per cm in the rhGH + A (vs untreated subjects) group, where no treated subject gained less than 4 cm.

We have not evaluated the effect of the treatment or of perceived height gain on the quality of life (QOL) of the participants. Not unexpectedly, all adolescents faced with a small height simply enjoyed the gain of extra height, and regretted only that the gain could not be greater. Our personal opinion is that QOL questionnaires and methodology are not specific enough to reflect people’s thoughts about their height accurately.

#### Safety issues

Concerns over long-term safety of rhGH have been recently revisited [81–84]. As summarized, rhGH has “an enviable track record of safety” [83]. Recent results have been reassuring [81, 85]. The current trial being maybe the shortest in duration of published studies, safety issues were only limited to short term tolerance.

The utilization and safety of aromatase inhibitors in male subjects were recently reviewed [86]. Lowering estradiol levels within the male physiological range is associated with an increase in levels of LH, FSH and testosterone [87, 88], as observed in the current study and found to be reversible with cessation of treatment. When treated with letrozole at the beginning of puberty, boys showed lower IGF1 levels than controls [89], an observation that was not replicated here under rhGH treatment. As reported [90], we observed a slight and reversible decrease in HDL-cholesterol, but none of the lipid values recorded during A administration could be considered even borderline-abnormal [91]. Letrozole-treated boys with ISS showed no loss of bone density [92, 93], but some mild vertebral deformities were observed in prepubertal boys with ISS or a delayed onset of puberty treated with letrozole for 2 years [71, 93]; we screened but did not observe any vertebral deformities in the studied adolescents. Again, the short duration of our trial does not allow us to draw any conclusion for mid or long term safety, and off label use of aromatase inhibitors for the treatment of short stature is currently not recommended outside a research setting.

## Conclusion

If the current results are confirmed, starting treatment of boys with ISS in late adolescence may have several advantages over the classical regimen of rhGH administration to healthy children predicted to be adults of very short stature. First, it allows more mature adolescents to participate in the decision of attempting rhGH treatment, a clear ethical advantage over treating children unable to participate to the decision. Secondly, the advanced epiphyseal fusion and near-ending growth trajectory allow a more accurate prediction of adult height than when it is attempted in earlier ages. Also, the shorter duration of administration may have a cost/benefit advantage.

In summary, a short administration of rhGH and A seems able to increase adult height in adolescents with ISS in their late stage of puberty, more than does rhGH alone, provided that epiphyseal plates are not completely fused at the knee and growth velocity is still significant at time of starting rhGH administration. However, because of the small size of the current trial, the lack of randomization versus untreated short adolescents and the short-term surveillance, we cannot recommend this off label treatment, which should remain in a clinical research setting. Larger RCTs will be needed to establish the cost/benefit ratio of rhGH and A administration when attempted in the late period of pubertal growth.

## Authors’ information

AR is pediatric endocrinologist, AL is professor of pediatric endocrinology, PB is professor of pediatric endocrinology and head of pediatric endocrinology, all three at Bicêtre hospital, Paris Sud University.

## Electronic supplementary material

Additional file 1: **List of parameters showing no significant correlation with height increase or height gain.** (DOC 28 KB)

Additional file 2: **Incidence of known secondary effects of A over the whole period of treatment in the two studied groups.** (DOC 30 KB)

Additional file 3: **Serum testosterone, gonadotropin levels and lipid parameters in adolescents treated with rhGH +Anastrazole.** (DOC 34 KB)
