# Erratum to: A randomized pilot trial of growth hormone with anastrozole versus growth hormone alone, starting at the very end of puberty in adolescents with idiopathic short stature

**DOI:** 10.1186/s13633-017-0043-0

**Published:** 2017-02-27

**Authors:** Anya Rothenbuhler, Agnès Linglart, Pierre Bougnères

**Affiliations:** Department of Pediatric Endocrinology, Bicêtre Hospital, Pôle I3E, AP-HP, Paris Sud University, 94275 Le Kremlin Bicêtre, France

## Erratum

This Erratum to our article [1] aims at making our randomization procedure more explicit, because our article did not explain the procedure in an appropriate manner, which could be misleading for future meta-analysis, and more generally for the readers. Moreover, this procedure can be of interest for clinicians who want to test two treatments of common practice.

Indeed, the comparison of the GH and [GH + anastrazole] treatments was based on the randomization of patients’ files to be analyzed, not on randomization of treatments themselves, as could be felt when reading our publication. Thus no treatment was dictated by randomization.

To perform our study, we randomly allocated a list of ordered numbers to treatment by GH or [GH + anastrazole]. For example, number 1 would be GH, number 2 [GH + anastrazole], number 3 [GH + anastrazole], number 4 GH, etc. This list kept by the PI was concealed to the physicians in charge of the patients. Children with very short stature who came in our clinic were simply given a number from 1 to 50 per order of arrival at the clinic. The choice of the clinicians in charge for using GH alone or [GH + anastrazole] was not influenced by these numbers. The use of anastrazole was inspired by publications showing a delay of epiphyseal maturation with this product.

At time of analysis, we used the numbers to retrieve only the patients’ files where randomly allocated treatment matched the treatment that had been actually decided by their doctor. In many cases, files did not match and were thus not used for the comparison, whereas the matching files allowed us to perform the randomized comparison. This original method allowed the study to combine the advantages of randomization while remaining entirely observational, and is best suited for comparing two treatments of current practice.
