# Supplementary material for: A randomized pilot trial of growth hormone with anastrozole versus growth hormone alone, starting at the very end of puberty in adolescents with idiopathic short stature
Source: Int J Pediatr Endocrinol. 2015 Feb 16;2015(1):4. doi: 10.1186/1687-9856-2015-4 (PMC4429943; doi:10.1186/1687-9856-2015-4)
Supplement: Supplementary file 1 — Additional file 1: List of parameters showing no significant correlation with height increase or height gain. (DOC 28 KB) [file 13633_2014_368_MOESM1_ESM.doc]

**Supplemental Table 1.** List of parameters showing no significant correlation with height increase or height gain.

| Knee Score |
| --- |
| Growth velocity at GH onset |
| GH dose |
| Bone age at hand  Age at GH onset  Time elapsed since HPV  Age at HPV |
| Testosterone  Delta IGF1 (SD) |
| IGF1 at GH onset (SD) |
