# Supplementary material for: A randomized pilot trial of growth hormone with anastrozole versus growth hormone alone, starting at the very end of puberty in adolescents with idiopathic short stature
Source: Int J Pediatr Endocrinol. 2015 Feb 16;2015(1):4. doi: 10.1186/1687-9856-2015-4 (PMC4429943; doi:10.1186/1687-9856-2015-4)
Supplement: Supplementary file 2 — Additional file 2: Incidence of known secondary effects of A over the whole period of treatment in the two studied groups. (DOC 30 KB) [file 13633_2014_368_MOESM2_ESM.doc]

**Suppl Table 2.** Incidence of known secondary effects of anastrozole over the whole period of treatment in the GH+A group.

|  | **Group treated**  **with GH alone** | **Group treated**  **with GH and anastrozole** |
| --- | --- | --- |
| Mood changes | None | Mild brief depressive feelings (1) |
| Neuropsychic symptoms | Mild insomnia (1); Transient weakness (1) | Nervousness (1) |
| Hot flashes | None | Hot flash (1) |
| Digestive symptoms | Vomiting (3) Constipation (1) | Vomiting (3) Stomach pain (1) |
| Skin | Acne (1) | Acne (1) |
| Joint symptoms | None | None |
| Pain | Back pain (2) Knee pain (1) | Back pain (3), Knee pain (1) |
