# Supplementary material for: A randomized pilot trial of growth hormone with anastrozole versus growth hormone alone, starting at the very end of puberty in adolescents with idiopathic short stature
Source: Int J Pediatr Endocrinol. 2015 Feb 16;2015(1):4. doi: 10.1186/1687-9856-2015-4 (PMC4429943; doi:10.1186/1687-9856-2015-4)
Supplement: Supplementary file 3 — Additional file 3: Serum testosterone, gonadotropin levels and lipid parameters in adolescents treated with rhGH +Anastrazole. (DOC 34 KB) [file 13633_2014_368_MOESM3_ESM.doc]

| **Supplemental Table 3.** Serum testosterone, gonadotropin levels and lipid parameters in adolescents treated with rhGH +Anastrazole. | | | |
| --- | --- | --- | --- |
|  | **Before onset of**  **treatment**  **(N=12)** | **At 1 year of treatment**  **(N=12)** | **6-12 months after end of treatment**  **(N=10)** |
| Testosterone (ng/dl) | 5.6 ± 0.9 | 8.4 ± 1.7 ** | 6.1 ±1.2 |
| LH (IU/L) | 4.3 ± 1.7 | 9.8 ± 3 ** | 4.6 ± 1.9 |
| FSH (IU/L) | 4.4 ± 1.9 | 11.6 ± 2.9 ** | 4.5 ± 1.8 |
|  |  |  |  |
| Total cholesterol (mmol/L) | 4.0 ± 0.9 | 3.8 ± 1.4 | 4.2 ± 0.8 |
| LDL-cholesterol (mmol/L) | 2.5 ± 0.7 | 2.6 ± 1.0 | 2.5 ± 08 |
| HDL-cholesterol (mmol/L) | 1.4 ± 0.2 | 1.3 ± 0.2 * | 1.5 ± 0.3 |
| Triglycerides (mmol/L) | 0.9 ± 0.3 | 0.9 ± 0.4 | 1.0 ± 0.4 |

** P < 0.01 * P < 0.05 in comparison with baseline values using a paired Student’s t test.
